# Is 2-deoxy-2-[^18^F]fluoro-D-glucose PET/CT acquisition from the upper thigh to the vertex of skull useful in oncological patients?

**Published:** 2014-12-19

**Authors:** B. Salvatore, M.G. Caprio, R. Fonti, D. D’Amico, F. Fraioli, M. Salvatore, L. Pace

**Affiliations:** 1 Consiglio Nazionale delle Ricerche–Istituto di Biostrutture e Bioimmagini, Napoli, Italy; 2 Dipartimento di Medicina e Chirurgia, Università degli Studi di Salerno, Italy; 3 Dipartimento ad attività integrate di Diagnostica Morfologica e Funzionale, Radioterapia, Medicina Legale. Università degli Studi di Napoli Federico II, Italy; 4 Fondazione SDN-IRCCS, Napoli, Italy

**Keywords:** PET/CT, whole-body acquisition, head, brain

## Abstract

**Aim:**

To assess whether performing routinely 2-deoxy-2-[^18^F]fluoro-D-glucose PET/CT (^18^FDG PET/CT) scan from the upper thigh to the vertex of skull is clinically relevant.

**Materials and Methods:**

3502 (1634 female; mean-age 60+16) consecutive patients undergoing ^18^FDG PET/CT were retrospectively analyzed. Patients were divided in 10 groups according to primary malignancy. Chi-square analysis was used to assess differences among proportions. A p value < 0.05 was considered significant.

**Results:**

^18^FDG PET/CT was positive in head district in 130/3502 (3,7%) patients. In all patients lesions were unknown before PET/CT examination. PET/CT showed 158 positive brain/head uptake in the 130 patients. The 158 lesions were localized in: brain (43/158; 27%), bone (52/158; 33%), lymph node (1/158; 0,6%), soft tissue (55/158; 35%) and other sites (7/158; 4,4%). According to each group, patients were positive in the head district in 1.0% for Gastrointestinal Cancer (7/690), 3.0 % for Genitourinary Cancer (3/101), 3.7 % for Haemathologic Cancer (59/1590), 2.7 % for Gynaecologic Cancer (3/112), 7.8% for Head-Neck-Thyroid and Parathyroid Cancer (26/331), 3.5% for Breast Cancer (7/200), 2.6% for Lung Cancer (7/271), 3.4% for Melanoma (2/59), 7.4% for Sarcoma (2/27), 11.6% for Unknown Primary Tumour (14/121).

**Conclusion:**

Our data show a relatively high incidence of brain/head lesion in patients with Unknown Primary Tumour.

## I. INTRODUCTION

2-deoxy-2-[^18^F]fluoro-D-glucose Positron emission tomography-computed tomography (^18^FDG PET/CT) is an established whole-body imaging technique used in clinical oncology practice. Whole-body ^18^FDG PET/CT (FDG-wb-PET/CT) has been used in differentiating and characterizing indeterminate lesions, in differentiating recurrent disease from therapeutic effects, in staging and evaluating the extent of disease, and in monitoring therapy in a variety of cancers [1–3].

Usually oncological ^18^FDG PET/CT scan is performed from the upper thigh to the base of skull, taking approximately 24–28 minutes, because most of FDG avid lesions are expected within this field of view. However, the detection of head region metastases may change staging and prognosis and thus the management of patients. Brain metastases are a relatively common complication of cancer and can affect up to 40% of patients, depending upon the primary histology, while skull metastases are less frequent [4–6]. The efficacy of FDG PET in describing cerebral metastases is controversial. The sensitivity of FDG PET in revealing cerebral metastases in patients with malignancy has been reported to vary from 68% to 82% when compared with anatomic imaging [4, 7]. This large range of sensitivity likely reflects two factors: first, the high level of physiologic cerebral FDG uptake which may interfere with the identification of them; and second, the inability of PET scanner to show lesions smaller than 6–10 mm. Therefore, many studies suggest that ^18^FDG PET/CT is suboptimal for the detection of cerebral and skull metastases and many centers do not routinely perform scanning of the head in whole-body FDG PET/CT scan [8–12]. However, ^18^FDG PET/CT scan including the head can discover unknown lesions in this anatomic district [4, 13–15], infact FDG-wb-PET/CT is the method of choice also for malignant head and neck tumors in both primary staging and restaging and its use, as a part of the therapeutic algorithm, results in a reduced number of unnecessary surgical procedures [16]. ^18^FDG PET/CT scan including the head may be relevant since all the main cross-sectional anatomical imaging techniques, such as CT and MR, are frequently performed not as total body examination, lacking the ability to detect and delineate intracranial and skull metastases.

Therefore, the aim of this study was to assess whether performing routinely ^18^FDG PET/CT scan from the upper thigh to the vertex of skull (FDG-wb-PET/CT) be clinically relevant, considering the length of scan time and exposure radiations.

## II. METHODOLOGY

### Patient population

A total of 3502 consecutive patients (1634 female; mean-age 60±16), undergoing FDG-wb-PET/CT scan for oncological purpose from May 2012 to November 2013, were retrospectively analyzed. All patients were divided according to their primary malignancy localization in 10 groups: Gastrointestinal Cancer, Genitourinary Cancer, Haemathologic Cancer, Gynaecologic Cancer, Head-Neck-Thyroid and Parathyroid Cancer, Breast Cancer, Lung Cancer, Sarcoma, Melanoma, Unknown Primary Tumor.

The present study was performed according to the declaration of Helsinki. All patients gave their informed consent for PET/CT examination. The present retrospective analysis did not require Institutional Review Board approval nor informed consent by national institution.

### FDG

FDG was synthesized using a Mini Trace GE cyclotron and CPCU automated chemistry module (Fx-FDG GE). 18Fluorine as fluoride was produced using a proton-neutron reaction on 95% enriched 18oxygen water. 18Fluorine-FDG was synthesized in the CPCU using the modified Hamacher synthesis. The product was delivered pure, sterile and in an injectable form.

#### Scan acquisition

All patients fasted 8 hours and underwent a blood glucose test examination before the injection of an average of 370 MBq of ^18^FDG via a cubital vein. None of the patients included in the study had serum glucose levels higher than 140 mG/dL. Patients were kept quiet and confortable for 60 min after the injection. No muscle relaxants were administered. Immediately before PET/CT scan acquisition, all patients were asked to empty their bladder.

Images were obtained on a Discovery LS (GE Medical Systems, Milwaukee, USA), an integrated system consisting in NXi PET scanner and Light Speed Plus four rows MDCT system.

PET/CT acquisition included a whole body acquisition: in supine position, vertex to upper thigh four-slice spiral CT scan with acquisition parameters of 140 kv, 80 mAs, 0,5 s/CT rotation, a pitch of 1.5, performed during normal breathing. This was followed by a PET acquisition of the same axial range for 4 min per cradle position. Patient’s head was locked to minimize head movement during imaging. ^18^FDG PET images were reconstructed with a 4.5 mm effective thickness. The CT data were used for attenuation correction and images were reconstructed using a standard iterative algorithm.

#### Image interpretation

^18^FDG-wb-PET/CT images were analyzed on a dedicated workstation (XELERIS, GEMS) in the coronal, sagittal and transaxial planes as well as 3-dimensional projections. Interpretation was based on evaluation of ^18^FDG uptake at the tumor site and surrounding sites. In particular, distinctly focal uptake higher than surrounding non diseased tissues and with a SUV max > 2.5 was considered as malignant, otherwise it was considered negative. Two experienced nuclear medicine physician, unaware of clinical as well as all other pertinent data, analyzed all patient studies.

#### Statistical analysis

A Chi-square analysis was used to assess differences among proportions. A p value < 0.05 was considered significant.

## III. RESULTS

Of the 3502 patients included in the study, 19.7% (690/3502) had Gastrointestinal Cancer, 2.8% (101/3502) Genitourinary Cancer, 45.4% (1590/3502) Hematologic Cancer, 3.2% (112/3502) Gynaecologic Cancer, 9.5% (331/3502) Head-Neck-Thyroid-Parathyroid Cancer, 5.7% (200/3502) Breast Cancer, 7.7% (271/3502) Lung Cancer, 1.7% (59/3502) Melanoma, 0.8% (27/3502) Sarcoma and 3.5% (121/3502) Unknown Primary Tumor.

**Fig. 1.**
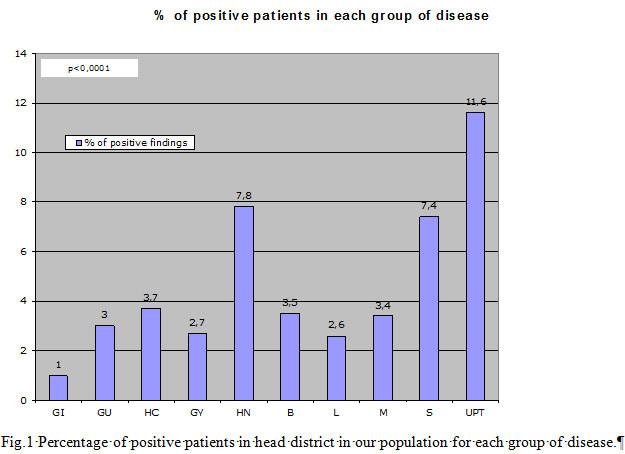
Percentage of positive patients in head district in our population for each group of disease.
GI = gastrointestinal cancerGU = genitourinary cancerHC = haemathologic cancerGY = gynaecologic cancerHN = head-neck-thyroid and parathyroid cancerB = breast cancerL = lung cancerM = melanomaS = sarcomaUPT = unknown primary tumor

^18^FDG-wb-PET/CT was positive in head district in 130/3502 (3.7%) of our population. None of the patients showed symptoms and/or clinical findings suggestive of such lesions. In particular, pathological uptake of ^18^FDG in the head district was found in 1.0% of Gastrointestinal Cancer patients (7/690), 3.0 % of Genitourinary Cancer patients (3/101), 3.7 % of Haemathologic Cancer patients (59/1590), 2.7 % of Gynaecologic Cancer patients (3/112), 7.8% of Head-Neck-Thyroid and Parathyroid Cancer patients (26/331), 3.5% of Breast Cancer patients (7/200), 2.6% of Lung Cancer patients (7/271), 3.4% of Melanoma patients (2/59), 7.4% of Sarcoma patients (2/27), 11.6% of Unknown Primary Tumour patients (14/121) (p< 0.0001) (Fig.1).

A total of 158 ^18^FDG-wb-PET/CT brain/head lesions were observed in the 130 patients. Of these 158 lesions, 27% (43/158) were cerebral, 33% (52/158) involved bone structures, 0.6% (1/158) involved lymph nodes, 35% (55/158) were localized in soft tissue and 4.4% (7/158) had other head location. Table 1 shows head lesions according to primary cancer disease. Brain lesions were more frequent in patients with lung and breast cancer while bone lesions and soft tissue localizations were more frequent in gastrointestinal and gynaecologic cancer and in haematological disease and melanoma respectively.

## IV. DISCUSSION

In this study a relatively low frequency (3.7%) of head abnormalities at ^18^FDG-wb-PET/CT was found. This finding is in agreement with that of a previous study [4], performed using only a PET scanner, suggesting that ^18^FDG-PET has a little impact in the investigation of cerebral metastases, with 3,9% of abnormal findings in head district.

However, in a particular subsets of disease, Unknown Primary Tumour a somewhat different percentage of head lesions (11.5%) was observed. Thus it seems to be appropriate to perform PET/CT including the whole head in this condition. On the other hand, it has been demonstrated that FDG-PET represents an accurate and useful test for the work-up of patients with suspected or proven intracranial metastases [17].

In a previous study [18] it has been recognized that among common malignant tumours that metastasize to the brain, lung cancer (41,6%), melanoma and breast cancer (8,3%) are more frequent. We found that brain lesions are more frequent in patients with breast and lung cancer as compared with the other neoplastic diseases studied. In particular in patients with lung cancer it has been demonstrated that there is a difference of metabolism in brain metastases from non-small-cell-lung cancer (NSCLC) and in those from small cell lung cancer (SCLC). One third of brain metastases from lung cancer are hypometabolic, but NSCLC was more frequently associated with hypermetabolic metastatic brain lesions than SCLC [19], in our group of patients we found only hypermetabolic lesions but we haven’t the histological correlation for each lesion.

Two studies performed in patients with melanoma showed that routine skull base to upper thigh acquisition is adequate for this group of patients and in particular PET is an extremely useful tool in the detection of metastatic melanoma, playing a primary role also in the staging of this disease to detect metastic lesions in particular in soft tissues [13, 20] as showed also by our study.

Kole A.C. et al. [15] showed that ^18^FDG-PET was able to identify the site of unknown primary tumor in 24% of patients unsuccessful studied by Conventional Imaging and in the present study PET was able to identify 1 brain lesion and 13 cervical lymph nodes, according to our results that showed among all types of cancer, PET/CT was positive in head district in 11,5% of patients with UPT, demonstrating the utility to perform a whole-body PET/CT including head in this subsets of patients. These data are also in agreement with two large meta-analysis of the performance of ^18^FDG-PET in the detection of Unknown Primary Tumor and they have demonstrated that ^18^FDG-PET could be useful in the staging of these patients [21, 22]. In the same way a recent study by Sebro et al. demonstrated that although the lesions found using a true whole-body FDG PET/CT scan, rarely change the stage, might have impact on clinical management [23].

Our findings demonstrated that soft tissue metastases detected with ^18^FDG-wb-PET/CT are in a higher percentage (35%) among all detected lesions. These results are quite similar to those of Nguyen NC. et al. [24] that concluded showing the detection of soft tissue metastases, higher in ^18^FDG-wb-PET/CT, may have a prognostic implications, providing more accessible biopsy sites and avoiding invasive procedures. Moreover Pfannenberg C. et al. [25] have demonstrated ^18^FDG PET/CT has higher sensitivity than MRI in detecting skin and soft tissue metastases, supporting the increasing role of ^18^FDG PET/CT in cancer patient management.

We acknowledge the limitations of our retrospective study. First of all we have not a histological correlation for each lesion. There are undoubtedly false positives findings, because ^18^FDG uptake and resulting increased tracer activity is not limited to neoplastic tissue and in the same way ^18^FDG PET/CT is suboptimal in detecting brain metastases due to the intense physiologic background uptake in the brain and the hypometabolic nature of some brain metastases. In addition ^18^FDG PET/CT from the vertex of skull to the upper thigh requires additional several minutes of image acquisition and also an additional radiation dose that can be useless for the all categories of patients.

## V. CONCLUSION

In conclusion our data support the importance of the inclusion of head district in the whole body ^18^FDG PET/CT scan only for patients with Unknown Primary Tumor where a faster diagnosis could avoid unnecessary extensive procedures at staging or follow-up.

## Figures and Tables

**Table 1.** Number and type of lesions for each cancer disease

**Group of disease**	**Brain lesions**	**Bone**	**Soft Tissue lesions**	**Lymph node**	Other*	**Total**
		**Metastases**				
**Gastrointestinal**	3 (43%)	4 (57%)				7
**Genitourinary**	1 (33%)	1 (33%)	1 (33%)			3
**Haemathologic**	27 (46%)	7 (10%)	31 (43%)	1 (1%)	6 (8%)	72
**Gynecologic**	1 (25%)	3 (75%)				4
**Head-neck**	10 (38%)	9 (27%)	14 (42%)			33
**Breast**	4 (57%)	2 (29%)	1 (14%)			7
**Lung**	4 (50%)	3 (38%)	1 (13%)			8
**Sarcoma**	1 (33%)	1 (33%)	1 (33%)			3
**Unknown Primary Tumour**	7 (50%)	7 (37%)	5 (26%)			19
**Melanoma**			1 (50%)		1 (50%)	2
**Total**	58 (38%)	37 (23%)	55 (35%)	1 (1%)	7 (4%)	158

* **= (lachrymal gland, orbit and ocular globe)**
